# Progressive Supranuclear Palsy With Post-traumatic Frontal Lobe Damage Mimicking Anti-IgLON5 Antibody Disease

**DOI:** 10.7759/cureus.99889

**Published:** 2025-12-22

**Authors:** Damla Ates Gulkok, Aiswarya Raj, Yakira Mishan, Jessica Bloom, Brittany Zaita, Robert Fekete

**Affiliations:** 1 Neurology, New York Medical College, Valhalla, USA

**Keywords:** chorea, frontal lobe injury, involuntary limb movement, progressive supranuclear palsy, psp

## Abstract

Phenotypic overlap between autoimmune encephalitis and movement disorders may rarely occur. We present a patient with progressive gait disturbance, vertical supranuclear gaze palsy, cognitive decline, and daytime somnolence, as well as extremity chorea. He was initially suspected of having autoimmune encephalitis. Antibody testing was negative in serum and cerebrospinal fluid. A fall leading to subdural hemorrhage and bilateral post-traumatic frontal lobe changes likely led to his bilateral upper and lower extremity involuntary movements. His mental status improved after lumbar puncture. His condition further improved on treatment with carbidopa levodopa 25/100 mg three times a day.

While the presentation of this case was suspicious for autoimmune encephalitis, the patient was diagnosed with progressive supranuclear palsy and traumatic encephalopathy with involuntary movements due to frontal lobe damage.

## Introduction

Phenotypic overlap between autoimmune encephalitis and movement disorders may rarely occur. Anti-IgLON5 antibody-mediated disease and progressive supranuclear palsy (PSP) may clinically mimic each other [1,2]. 

We present a patient with progressive gait disturbance, vertical supranuclear gaze palsy, and cognitive decline consistent with PSP, with an enigmatic clinical examination finding of chorea, which led to concerns for autoimmune encephalopathy. This case highlights the phenotypic overlap between PSP and anti-IgLON5 disease. It was ultimately determined that the patient’s unusual clinical presentation was due to the combination of PSP and traumatic encephalopathy.

PSP is one of the tauopathies and a clinicopathologic entity marked by vertical supranuclear gaze palsy, pseudobulbar palsy, symmetric bradykinesia, frontal-subcortical cognitive dysfunction, gait impairment, and postural instability. In contrast, anti-IgLON5 disease, associated with antibodies against IgLON5, a neuronal cell-adhesion protein, typically presents with combined non-rapid eye movement (REM)/REM parasomnias (stridor and obstructive sleep apnea), bulbar dysfunction (dysarthria, dysphagia, vocal-fold paresis, or episodic respiratory failure), and gait disturbance [2]. Notably, both conditions feature prominent gait and movement abnormalities, which can often confound the differential diagnosis. 

This article was previously posted to the Research Square preprint server on October 12, 2025 [3].

## Case presentation

The patient was a 76-year-old Hispanic male who was transferred to our hospital for evaluation for normal pressure hydrocephalus. There was no past medical history. There was no family history of neurological disorders. 

**Figure 1:**
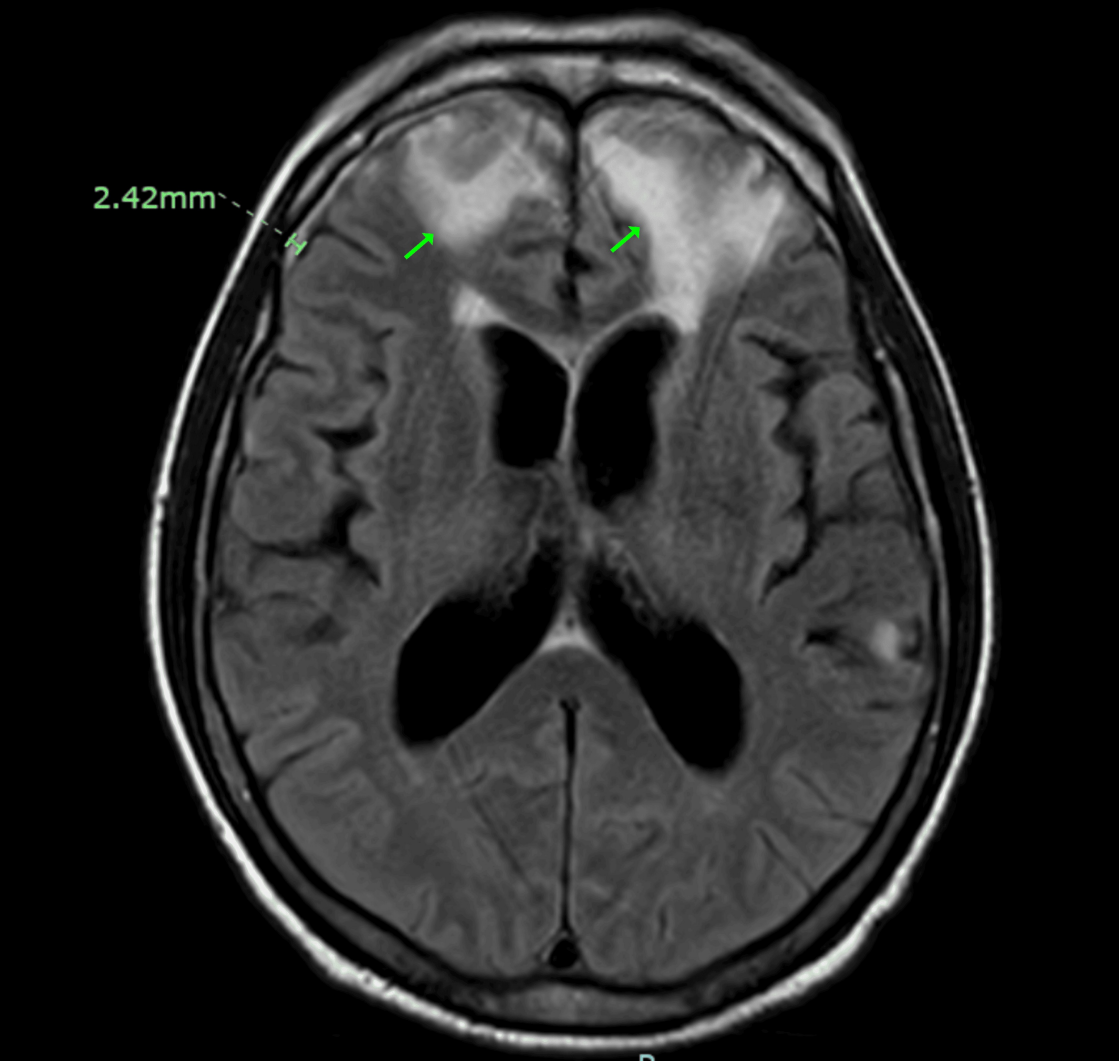
MRI brain with bilateral frontal lobe hyperintensities and right subdural hemorrhage. MRI T2 Fluid Attenuation Inversion Recovery (FLAIR) hyperintensities in bilateral frontal lobes (arrows) as well as a 2.42 mm right convexity subdural hemorrhage (bracket).

He was in his usual state of good health until about six months prior to his presentation, at which time he developed subacute, progressive memory loss and gait disturbance with imbalance. The imbalance led to a fall on the stairs in which he hit his occiput and had a contrecoup contusion and hemorrhage of the bilateral frontal lobes one month prior to his presentation. Imaging at this time was significant for T2 Fluid Attenuation Inversion Recovery (FLAIR) hyperintensities in bilateral frontal lobes as well as a 2.42 mm right convexity subdural hemorrhage as seen on MRI in Figure 1. Following an admission for his traumatic brain injury, he went to inpatient rehabilitation. His rehabilitation course was complicated by progressive drowsiness. Lumbar puncture was performed, which his family reports resulted in him becoming more awake and cooperative. Hence, post-traumatic hydrocephalus was considered and he was transferred to our hospital for higher level of care. 

At the time of transfer to our hospital, he had waxing and waning mental status with about five-minute episodes of depressed mental status with closed eyes, not following verbal commands, but withdrawing to noxious stimuli and increased tone in the upper extremities bilaterally, which was bordering on catatonic. He did not have any verbal output and did not follow verbal commands during these episodes. Lifting of either extremity by the examiner led to him holding up that extremity for about three seconds before slowly dropping it back down. He was previously evaluated by the psychiatry department at the sending institution and catatonia was considered. He was given a benzodiazepine trial (Valium 5 mg single dose) with the family reporting absence of improvement. He was also noted to have chorea of both hands and feet, which was moderate continuous.

He subsequently had five-minute episodes of clearer mental status during which he had open eyes and he could intermittently follow one-step commands such as performing finger taps and finger-to-nose testing. He was also oriented to self and hospital. He was able to provide brief, up to two-word answers to verbal questions. There was a paucity of spontaneous speech. 

For the frontal release signs, the glabellar sign was positive, the palmomental sign was negative bilaterally, and the orbicularis oris (snout) reflex was negative. The rest of the examination was the same. 

On cranial nerve examination, during the depressed mental status episodes he did not have horizontal or vertical pursuit but he did have normal oculocephalic response horizontally and vertically. During improved mental status episodes as well as after repeat lumbar puncture at our hospital, he had apraxia of horizontal gaze on verbal command, but he could perform horizontal pursuit towards the right and left. He also would spontaneously have slow saccadic movements horizontally to a vertically moving stimulus. He could not perform vertical gaze movements on command. There was no vertical upward gaze on pursuit. There was severely diminished downward gaze on pursuit. He was also noted to have a decreased blink rate and at times an eyelid-opening apraxia. 

During all levels of mental status, he had diminished but still present horizontal optokinetic kinetic nystagmus (OKN). There was an absence of upward and downward OKN. There was horizontal nystagmus while the patient was provided upward and downward moving stripe stimuli during vertical OKN testing. There was a normal oculocephalic response with vertical and horizontal head tilt in all directions. 

Electroencephalography (EEG) was negative for epileptiform waves. He received a lumbar puncture with an opening pressure of 10 cm H_2_O, glucose normal at 51 mg/dL, 4 white blood cells per mm^3^, 2 red blood cells per mm^3^ and mild elevation in protein to 46 mg/dL (normal 15-45 mg/dL) (Table 1). Cerebrospinal fluid (CSF) lactate dehydrogenase was normal at 24 units/L. CSF analysis showed a normal proportion of 89% lymphocytes as well as 0% lymphocytes and low monocytes at 11% (normal 16-56%). CSF Gram stain and bacterial cultures were negative. Further metabolic workup for his decline in mental status was negative (Table 1). Paraneoplastic autoimmune testing in serum and CSF was negative, including anti-IgLON5 and anti-Ma2 antibodies (Table 2).

**Table 1:** Cerebrospinal Fluid (CSF) and Serum Biochemical Studies Results of CSF and serum biochemical studies with normal ranges. CEA: carcinoembryonic antigen, CA 19-9: cancer antigen 19-9, PMN: polymorphonuclear lymphocytes

CSF Lab Studies	Results	Normal Values Range () and Units
CSF Biochemistry		
Glucose	51	
Protein	46	(15 - 45) mg/dL
Lactate dehydrogenase (LDH)	24	<40 U/L
CA 19-9	< 6	< 6 U/mL
CSF Cell Count with Differentials		
WBC	4	(0 - 5) per mm^3^
RBC	2	(0) per mm^3 *^Tube sample #2
PMN	0	(0 - 7) %
Lymphocytes	89	(28 - 96) %
Monocytes	11	(16 - 56) %
CSF Cultures and Microbiology		
Gram Stain	NEG	
Bacterial Culture	NEG	
Serum Studies		
Biochemistry		
Copper	120	(73 - 129) mcg/dL
Ferritin	356.1	(18 - 370) mcg/dL
Total Iron	38	(60 - 160) mcg/dL
Total Iron Binding Capacity (TIBC)	160	(265 - 375) mcg/dL
Unsaturated Iron Binding Capacity (UIBC)	122	(136 - 286) mcg/dL
Iron Percent Saturation	24	(18 - 56) %
Immunology and Protein Studies		
IgG4	14.1	(3.9 - 86.4) mg/dL
Serum Tumor Markers		
CEA	1.5	(0 - 10) ng/mL
CA 19-9	14	<35 U/mL

**Table 2:** Autoimmune Studies in Cerebrospinal Fluid (CSF) and Serum. Results of autoimmune studies in CSF and serum. CBA: cell binding assay, IFA: immunofluorescence, RIA: radioimmunoassay, ELISA: enzyme-linked immunosorbent assay, Ab: antibodies.

CSF Autoimmune Studies	Methodology	Results	Normal Range Values () and Units
AMPA-R Ab	CBA	NEG	NEG
Amphiphysin Ab	IFA	NEG	NEG
Anti-Glial Nuclear Ab, Type I	IFA	NEG	NEG
Anti-Neuronal Nuclear Ab, Type I-II-III	IFA	NEG	NEG
CASPR2-IgG	CBA	NEG	NEG
CRMP5-IgG	IFA	NEG	NEG
GABA-B-R Ab	CBA	NEG	NEG
GAD-65 Ab Assay	RIA	0	<= 0.02 nmol/L
GFAP	IFA	NEG	NEG
LG1-IgG	CBA	NEG	NEG
mGluR1 Ab	IFA	NEG	NEG
NIF	IFA	NEG	NEG
NMDA-R Ab	CBA	NEG	NEG
Purkinje Cell Cytoplasmic Ab Type I-II	IFA	NEG	NEG
SEPTIN-7	IFA	NEG	NEG
Neurochondrin	IFA	NEG	NEG
TRIM-46 Ab	IFA	NEG	NEG
DPPX Ab	CBA	NEG	NEG
IgLON5	CBA	NEG	NEG
Ma2	ELISA	NEG	NEG
Serum Autoimmune Studies	Methodology	Results	Normal Range Values and Units
AMPA-R Ab	CBA	NEG	NEG
Amphiphysin Ab	IFA	NEG	NEG
Anti-Neuronal Nuclear Ab, Type I-II-III	IFA	NEG	NEG
CASPR2-IgG	CBA	NEG	NEG
CRMP5-IgG	IFA	NEG	NEG
GABA-B-R Ab	CBA	NEG	NEG
GAD-65 Ab Assay	RIA	0	<= 0.02 nmol/L
GFAP	IFA	NEG	NEG
LG1-IgG	CBA	NEG	NEG
mGluR1 Ab	IFA	NEG	NEG
NIF	IFA	NEG	NEG
NMDA-R Ab	CBA	NEG	NEG
Purkinje Cell Cytoplasmic Ab Type I-II	IFA	NEG	NEG
DPPX Ab	CBA	NEG	NEG
IgLON5	CBA	NEG	NEG
AGNA-1	IFA	NEG	NEG
Ma2	ELISA	NEG	NEG

**Figure 2:**
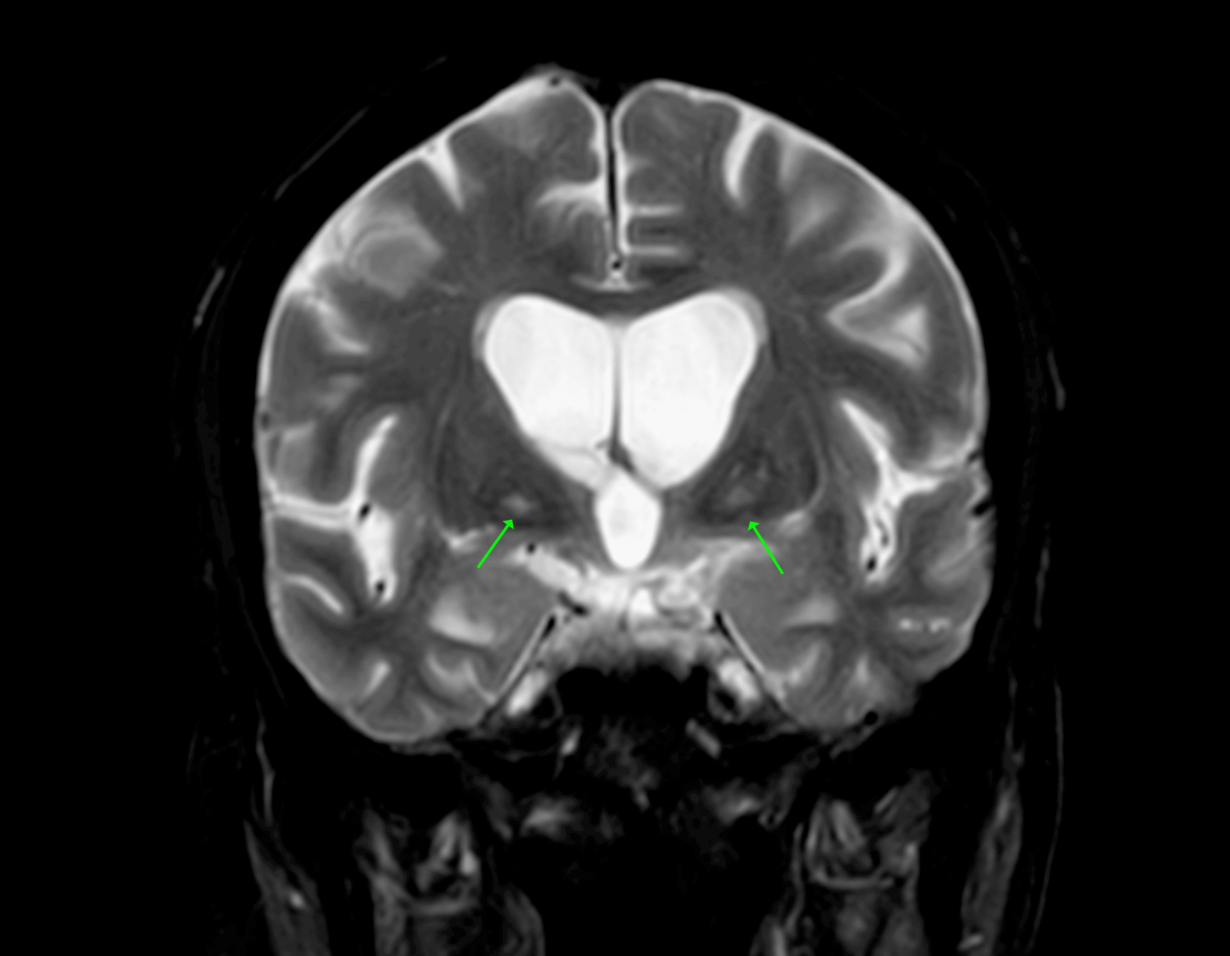
"Eye-of-the-tiger-like" appearance. Coronal MRI T2 Fluid Attenuation Inversion Recovery (FLAIR) image demonstrating "eye-of-the-tiger-like" appearance (arrows).

**Figure 3:**
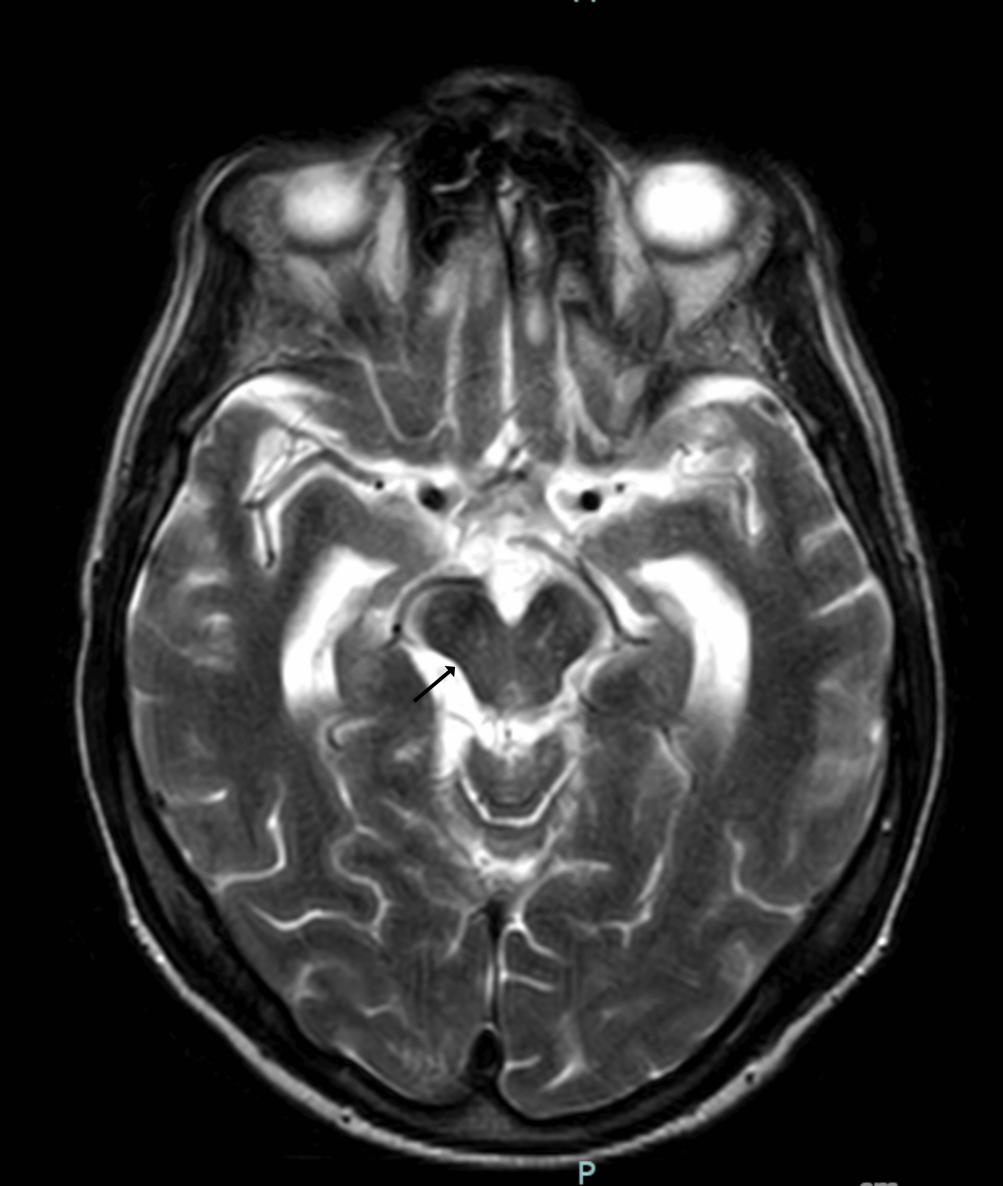
Midbrain atrophy. T2-weighted Turbo Spin Echo (TSE) axial MRI brain image showing midbrain atrophy (arrow).

MRI brain showed "eye-of-the-tiger-like" appearance (Figure 2) and midbrain atrophy (Figure 3) [4]. 

After this lumbar puncture, he remained in a clearer mental state continuously. He was alert and oriented to person and place with paucity of verbal output. Three-word responses to verbal questions were present and he was following one-step commands with eyes mostly open, but intermittently manifesting apraxia of eyelid opening and horizontal gaze apraxia.

At this time, the patient could perform only the first step of the Luria sequence with the right hand. He could not perform any steps of the Luria sequence with the left hand. He had continuous stereotypic hand-wringing movements bilaterally and continuous chorea of the feet. The patient was more attentive and engaging, however chorea remained present at a moderate level after the administration of carbidopa/levodopa 25/100 mg tablets three times a day. He had improvement of upper extremity rigidity from moderate to mild post-carbidopa/levodopa initiation.

## Discussion

IgLON plays an important role in the process of neuronal adhesion, neurogenesis, and neuroplasticity, and may play a role in maintaining the blood-brain barrier [4]. Anti-IgLON5 disease can present with nonspecific symptoms such as shortness of breath during sleep, cognitive decline, gait disturbance, and autonomic nervous system dysregulation [4]. Anti-IgLON5 disease not only has antibodies against the IgLON5 protein but also shows a deposition of hyperphosphorylated tau in the tegmentum of the brainstem and hypothalamus, leading to progressive neurodegeneration similar to other tauopathies such as PSP [2]. Patients with gaze palsy and postural instability diagnosed with PSP can develop these symptoms as a result of anti-IgLON5 [4,5].

IgLON5 has a variable presentation, with sleep disorder, bulbar symptoms, gait abnormalities, and cognitive dysfunction being the most prevalent. Other common representations include oculomotor abnormalities with vertical or horizontal gaze palsy, nystagmus, and ptosis. Some patients have been reported to have dysautonomia, cerebellar signs, and muscle weakness. Almost any movement disorder has been reported, ranging from hyperkinetic to hypokinetic. In fact, some studies have identified a few clinical phenotypes that feature sleep disorder predominance, bulbar symptoms, PSP-like syndrome, and cognitive disorders with or without chorea. In this patient, he did present with an altered level of consciousness resembling sleep attacks of anti-IgLON5 disease [6].

While the oculomotor features of PSP and IgLON5 disease show great overlap, a lack of saccadic intrusions and square wave jerks has been found to be more common in anti-IgLON5 than in PSP, which may be a result of the involvement of the cerebellum in PSP but not in IgLON5 disease [7]. In contrast, supranuclear gaze palsy, a defining feature of PSP, is not always present in anti-IgLON5 disease. 

This patient presented with a complex combination of symptoms, as he had a six-month history of memory disorder and gait imbalance in keeping with the PSP phenotype seen on examination. This ultimately resulted in his fall and traumatic brain injury. 

The patient’s frontal lobe injury, as seen in Figure 1, and possible post-traumatic hydrocephalus led to akinetic mutism with closed eyes and no verbal output. Repeat lumbar puncture improved his mental status. Absent vertical OKN with normal oculocephalic reflex is consistent with supranuclear gaze palsy. Midbrain atrophy in Figure 3 is a classic finding in PSP. However, the chorea of his lower limbs is atypical for PSP and raised concern for anti-IgLON5 disease. 

The combination of PSP clinical features, including rigidity, gait disturbance, and classic absence of vertical OKN, together with extremity chorea, is an unusual presentation of anti-IgLON5 antibody disease. Midbrain atrophy is a feature of either anti-IGLON5 antibody disease or PSP [2]. The "eye-of-the-tiger" sign is classically associated with pantothenate kinase-associated neurodegeneration (PKAN) and other diseases in the neurodegeneration with brain iron accumulation (NBIA) family. The patient's clinical presentation is not consistent with PKAN/NBIA. The "eye-of-the-tiger" sign is only rarely reported in PSP via individual case reports [3]. This sign has not been demonstrated in anti-IgLON5 antibody disease. The "eye-of-the-tiger-like" appearance in Figure 2 is not likely to be correlated with our patient's clinical presentation. 

## Conclusions

The combination of PSP clinical features, including rigidity, gait disturbance, and classic absence of vertical OKN, together with extremity chorea, raised concern for anti-IgLON5 antibody disease. Antibody testing was negative and the unusual phenotype was ascribed to bilateral frontal lobe post-traumatic changes in addition to PSP symptomatology. Midbrain atrophy is a feature of either anti-IGLON5 antibody disease or PSP. 
